# Reed-Sternberg cells in Hodgkin's lymphoma present features of cellular senescence

**DOI:** 10.1038/cddis.2016.185

**Published:** 2016-11-10

**Authors:** J Gopas, E Stern, U Zurgil, J Ozer, A Ben-Ari, G Shubinsky, A Braiman, R Sinay, J Ezratty, V Dronov, S Balachandran, D Benharroch, E Livneh

**Affiliations:** 1 The Shraga Segal Department of Microbiology Immunology and Genetics, Faculty of Health Sciences, Ben Gurion University of The Negev, Beer Sheva 84105, Israel; 2 Department of Oncology, Soroka University Medical Center, Beer Sheva 84105, Israel; 3 Flow Cytometry Unit, Hematology Laboratory and Institute of Hematology, Beer Sheva 84105, Israel; 4 Department of Pathology, Soroka University Medical Center, Beer Sheva 84105, Israel; 5 Blood Cell Development and Function Program, Fox Chase Cancer Center, Philadelphia, PA 19111, USA

## Abstract

Hodgkin's Lymphoma (HL) is one of the most prevailing malignancies in young adults. Reed–Sternberg (RS) cells in HL have distinctive large cell morphology, are characteristic of the disease and their presence is essential for diagnosis. Enlarged cells are one of the hallmarks of senescence, but whether RS cells are senescent has not been previously investigated. Here we show that RS cells have characteristics of senescent cells; RS cells in HL biopsies specifically express the senescence markers and cell cycle inhibitors p21^Cip1^ and p16^INK4a^ and are negative for the proliferation marker Ki-67, suggesting that these cells have ceased to proliferate. Moreover, the RS-like cells in HL lines, stained specifically for senescence-associated *β*-galactosidase (SA-*β*-gal). Oxidative stress promoted senescence in these cells as demonstrated by their staining for p21^Cip1^, p16^INK4a^, p53 and *γ*H2AX. Senescent cells produce copious amounts of inflammatory cytokines termed ‘senescence-associated secretory phenotype' (SASP), primarily regulated by Nuclear Factor *κ*B (NF-*κ*B). Indeed, we show that NF-*κ*B activity and NF-*κ*B-dependent cytokines production (e.g., IL-6, TNF-*α*, GM-CSF) were elevated in RS-like cells. Furthermore, NF-*κ*B inhibitors, JSH-23 and curcumin reduced IL-6 secretion from RS-like cells. Thus, defining RS cells as senescent offers new insights on the origin of the proinflammatory microenvironment in HL.

Hodgkin's lymphoma (HL), a B-cell originated malignancy of the immune system, is one of the most frequent lymphomas in the Western world, with an annual incidence of about three cases per 100 000 persons. This lymphoid malignancy involves peripheral lymph nodes and can also affect organs such as liver, lung, and bone marrow. HL is one of the most prevailing malignancies in young adults, with a second peak in the elderly.^1^ Although the rate of cure is high, about 20–35% of patients relapse, and about half of them eventually die of the disease or treatment-related late toxicities and secondary malignancies.^2^ Conceptually novel treatment strategies are thus needed, particularly for this category of patients.

Interestingly, the malignant cells are quite rare within the lymphoid mass, and usually account for only about 1-5% of all cells in the tumor tissue. In classical HL (cHL), the tumor cells are composed of Hodgkin (H) and Reed–Sternberg (RS) cells, representing the small mononucleated and large mono- or multinucleated subtype, respectively, and collectively termed Hodgkin and Reed-Sternberg (HRS) cells.^3^ RS cells exhibit a diameter of up to 100 *μ*m, whereas the proliferating mononuclear Hodgkin (H) cells, prominent in HL cell lines, is considerably smaller with a diameter of 20–30 *μ*m.^4^ Although Hodgkin and Reed–Sternberg cells (HRS) cells are derived from mature B cells, they have largely lost their B cell phenotype, are often large and multi-nucleated, and show a very unusual co-expression of markers of various hematopoietic cell types.^5^ Importantly, HRS cells show deregulated activation of multiple signaling pathways, including several proinflammatory cascades such as Nuclear Factor *κ*B (NF-*κ*B) and Jak/STAT pathways that are thought to contribute to the pathogenesis of HL.^3^ How HRS cells produce proinflammatory signals to recruit additional cell types into the diseased lymph node and contribute to tumor development is not known, and represents a significant gap in our knowledge of HL progression, with important clinical ramifications.

Cellular senescence is a terminal cell cycle arrest program, which is engaged in response to progressively shortened telomeres at the end of a cell's proliferative lifespan (known as replicative senescence),^6^ or may be acutely induced by activated oncogenes or DNA-damaging chemotherapy (termed premature senescence),^7, 8, 9^ thus reflecting a failsafe mechanism against imminent cellular insults. Senescent cells remain viable, exhibit a typical enlarged morphology and present a characteristic gene expression profile, including elevated expression levels of the cell cycle inhibitors p16^INK4a^ and p21^Cip1^. These cells express a senescence-associated *β*-galactosidase (SA-*β*-gal) activity, partly reflective of increased lysosomal mass. Senescent cells have been shown to secrete a plethora of factors, of largely NF-*κ*B-driven inflammatory cytokines chemokines and immune modulators and upregulate enzymes that degrade extracellular matrix, changes collectively called the senescence-associated secretory phenotype (SASP),^10, 11, 12^ which may, at least in some cell types, help to reinforce the senescence arrest.^13, 14^ Thus, although senescence functions as an antiproliferative program, capable of limiting tumorigenesis, senescent cells can also promote an inflammatory microenvironment that stimulates tumor progression.

RS cells represent the most prominent HRS-cell subtype in biopsies specimens and are defined as differentiated-end state of HRS cells, having a pivotal role in the interaction with the tumor microenvironment.^15, 16, 17^ Thus, the large cell morphology of RS cells is well established in this disease and is essential for diagnosis, but whether these cells are senescent has not been previously investigated. Here we show that a subpopulation the malignant HRS cells within classical HL show characteristics of the senescence phenotype and are thus responsible, at least in part, for the proinflammatory milieu seen in these tumors.

Here we demonstrate that RS cells in HL tumor biopsies and large RS-like cells in HL derived cell lines express markers of senescence, and can be induced to senesce in culture by oxidative stress. Moreover, large RS-like cells show enhanced NF-*κ*B activity and cytokines production, which can be suppressed by NF-*κ*B inhibitors. Defining RS cells as senescent enables better understanding of the molecular and cellular mechanisms underlying this malignancy providing new insights on the origin of the proinflammatory microenvironment in HL.

## Results

### HL tumor biopsies contain RS cells expressing senescence markers

**Figure 1:**
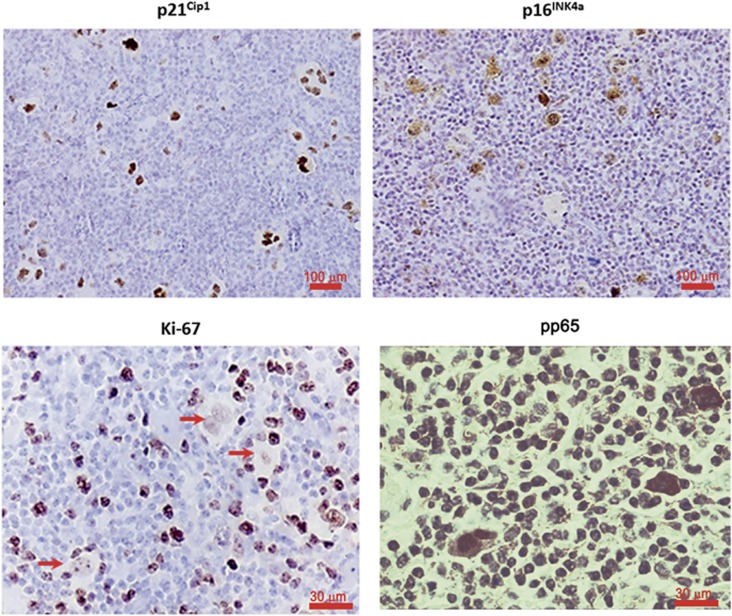
Expression of senescence markers in biopsies of classic Hodgkin's lymphomas. Patients' biopsies show expression of the cell cycle inhibitors p21^Cip1^ and p16^INK4a^ and NF-*κ*B phospho-p65 in RS cells. RS cells are not stained by the cell proliferation marker Ki-67 (arrows)

HL biopsies exhibit characteristic large RS cells. Most frequently, these cells are multinucleated, suggesting that they are arrested, and will not undergo normal replication. The large cell morphology is well established in HL, which is also considered as one of the consistent characteristic features of senescence. A pilot study on patients' biopsies of classical HL showed staining with HL markers CD30 and CD15 (Table 1). The cell cycle inhibitors and senescence markers, p16^INK4a^ and p21^Cip1^, as well as the NF-*κ*B subunit p65, were detected in most biopsies tested. Representative biopsies with p16^INK4a^ and p21^Cip1^ and phospho-p65 are depicted (Figure 1). Staining of p16^INK4a^ and p21^Cip1^ was mostly present in the large RS cells but not in the small proliferating Hodgkin cells. We have also found that RS cells in most biopsies were negatively stained for the proliferation marker Ki-67, supporting the notion that these cells are not replicating (Figure 1).

### Characterization of senescence markers in large RS-like cells in HL derived cell lines

**Figure 2:**
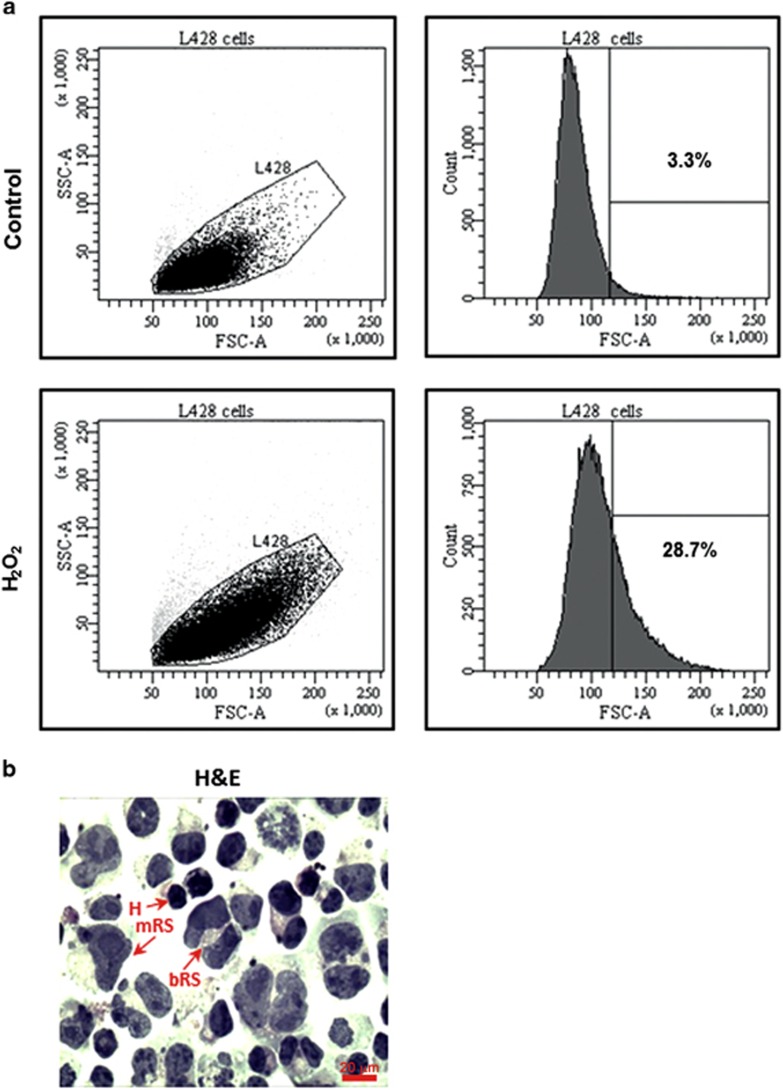
The number of large RS cells is increased by oxidative stress. Cells were treated with 50 *μ*M H_2_O_2_ for 2 h followed by 96 h incubation in growth medium. Cells of different size were determined by flow cytometry measurement of the cell forward-angle light scatter (FSC-A). To discriminate between large and small cells, the cutoff value equal to 120 AU (arbitrary units) was determined to correspond to the mean number of morphologically detected large cells in control cell cultures. (**a**) The results of a representative experiment (out of four independent reproducible experiments) depict the percentage values of large cells in control (3.3%) and in H_2_O_2_-treated cultures (28.7%) of L428 cells. (**b**) H_2_O_2_- treated cells were cytospined and stained by H&E. Arrows: H, Hodgkin small cells, mRS, mononuclear large RS and bRS, binuclear large RS cells

The HL-derived L428 and KHM2 cells exhibit mostly small proliferating (20–30 *μ*m) but also a small subpopulation of large RS-like cells (60–100 *μ*m). We show that this large RS-like subpopulation comprises 4.25+1.11% of cells in L428 cells, which was increased by about sevenfold (28.25+3.12%) using senescence inducing agents such as H_2_O_2_ (Figure 2a) or DNA-damaging agents such as etoposide (data not shown). The presence and morphology of RS-like cells is shown in Figure 2b.

**Figure 3:**
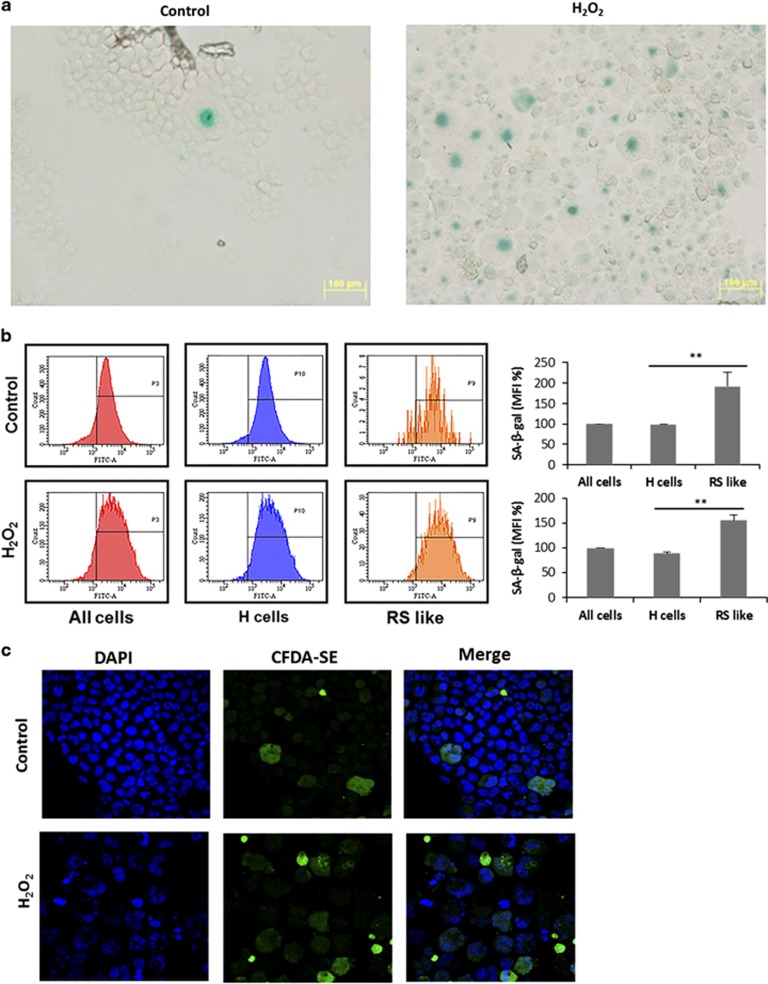
The senescence marker *β*-gal is exhibited by large RS cells. (**a**) Oxidative stress increased the number of large RS cell stained by *β*-Gal. Cells were treated with 50 *μ*M H_2_O_2_ for 2 h followed by 96 h incubation in growth medium and adhered on slides by cytospin centrifugation, and *β*-gal was detected as described in Materials and Methods. (**b**), Large RS cells express increased levels of *β*-gal. Control and H_2_O_2_-treated L428 cells were stained by the *β*-gal substrate C_12_FDG and the fluorescence of unsorted (all cells) and sorted small (H) and large (RS like) cells was analyzed by flow cytometry. A representative experiment is depicted. The bar graph represents the mean fluorescence intensity (MFI, %) and ±S.D. of three independent experiments. Significance of **P<0.05 was determined by *t*-test. (**c**), Large RS-like cells have limited proliferation capacity. The florescent dye CFDA-SE is retained in non-dividing cells and is diluted out in proliferating cells. Cells were labeled by CFDA-SE and fluorescence was detected after 96 h as described in Materials and Methods. The cells were photographed using Olympus confocal microscope 96 h after labeling ( × 600)

The senescence marker SA-*β*-gal stained specifically the large RS cells in L428 cells in control non-treated (6±2%) and H_2_O_2_ treated (31±8%) cells (Figure 3a). Similar results were obtained in KMH2 cells (data not shown). Our results and those of others indicate that the large RS-like cells are spontaneously formed in cell culture.^3^ Staining with the fluorescent *β*-gal substrate C_12_FDG followed by FACS analysis demonstrated SA-*β*-gal fluorescence in RS-like cells that was increased by oxidative stress (Figure 3b). The fact that these cells are not dividing was further confirmed by labeling the cells with the florescent dye CFDA-SE that remains in non-dividing cells and is diluted out in proliferating cells. We show that fluorescence was diminished in small dividing cells but retained in the large RS-like cells. The percentage of fluorescent positive cells was 4±2 in non-treated cells and 36±9 in H_2_O_2_ treated cells (Figure 3c).

**Figure 4:**
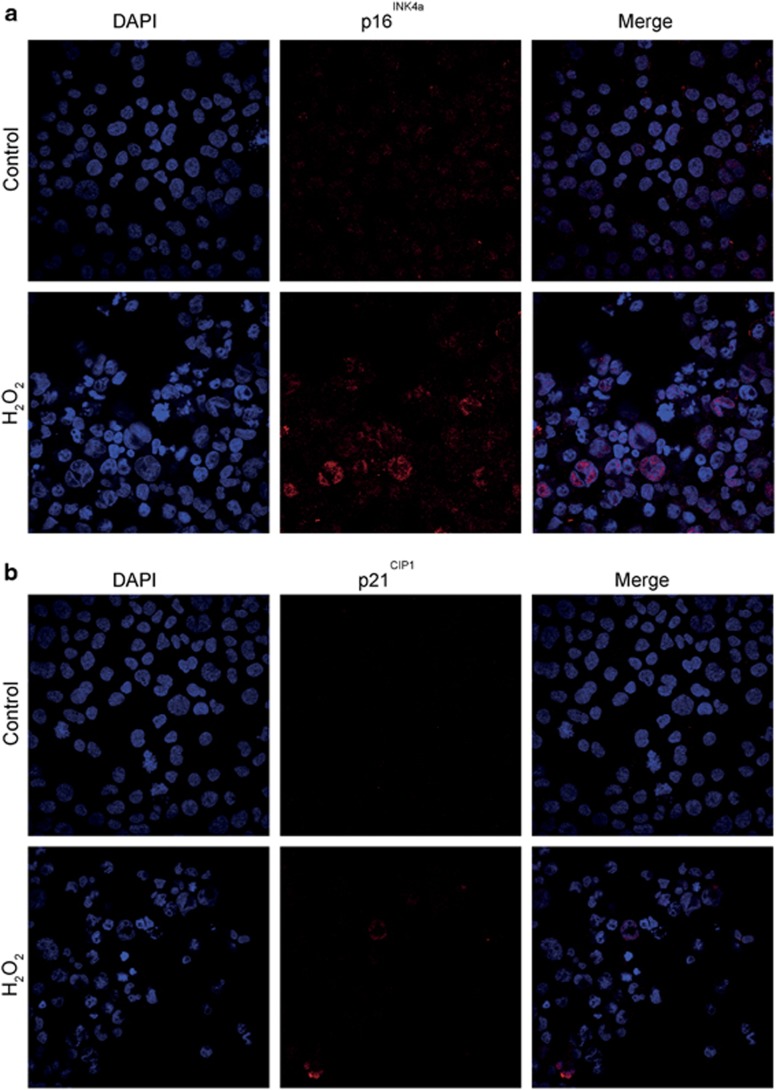
The senescence markers p16^INK4a^ and p21^Cip1^ are expressed in RS-like cells. Cells were treated with 50 *μ*M H_2_O_2_ for 2 h followed by 96-h incubation in growth medium. The cells were adhered on slides by cytospin centrifugation and stained by immunofluorescence with specific antibodies to p16INK4a (**a**) and p21Cip1 (**b**) as described in Materials and Methods. Cells were photographed using Olympus confocal microscope ( × 600)

Additional senescence markers were also demonstrated in H_2_O_2_ treated and non-treated L428 cells by immunofluorescence. As shown in Figure 4a, p16^INK4a^ was observed only in large RS and not in small cells (100% of non-treated and 93±3% of H_2_O_2_-treated large RS cells). Similarly, nuclear p21^Cip1^ was mostly stained in the H_2_O_2_ treated large RS-like cells (82±5%), and much less (10±5%) in the small cell subpopulation. L428 cells express wild-type p53.^24^ Notably the large RS-like cells expressed high levels of p53 (Supplementary Figure S1). The changes in these phenotypes confirmed that cellular senescence accompanied by gross changes in chromatin composition was induced by H_2_O_2_. The fact that these cells were under oxidative stress was confirmed by increased *γ*H2AX staining, a marker of double-strand DNA breaks (Supplementary Fig. S1).

### Large RS-like cells exhibit increased inflammatory cytokine secretion

**Figure 5:**
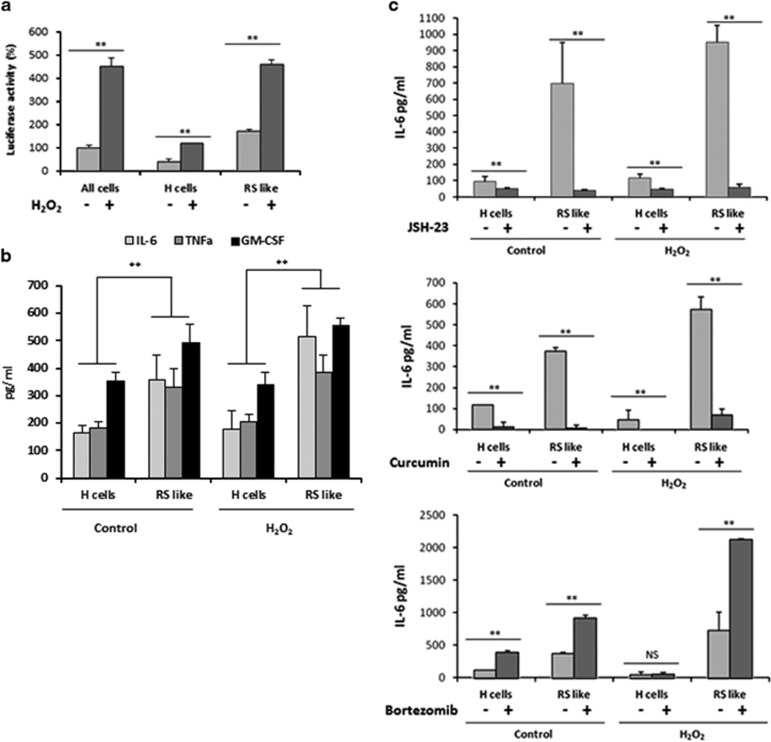
RS-like cells exhibit high NF-*κ*B activity. (**a**) NF-*κ*B luciferase activity was determined in non-sorted L428 cells and in FACS-sorted H and RS cells in the presence or absence of H_2_O_2_ as described in Materials and Methods. (**b**) RS like cells are the main source for cytokine secretion. Control and H_2_O_2_ treated cells were FACS sorted as in (**a**). Supernatants were collected 24 h after plating followed by ELISA. Large RS cells expressed higher levels of IL-6, GM-CSF and TNF-*α* compared with H cells (calculated for each individual cytokine). (**c**) NF-*κ*B is a modulator of IL-6 production in RS cells. Control and H_2_O_2_-treated cells were FACS sorted as in **a**. The specific NF-*κ*B inhibitor JSH-23 (100 *μ*M), Curcumin (30 *μ*M) and Bortezomib (10 nM), were added to sorted cells and supernatants were collected after 24 h. IL-6 was determined in triplicates by ELISA and normalized to 10^6^ cells. The experiments were repeated three times and mean and ±S.D. were calculated. Significance of at least **P<0.05 was determined by *t*-test

NF-*κ*B was previously shown to be a master regulator of cytokine secretion in senescence.^25^ Therefore, we have directly investigated the role of NF-*κ*B activation in H and large RS-like cells utilizing L428 cells harboring the NF-*κ*B luciferase reporter plasmid.^18^ NF-*κ*B activity was examined in H and large RS-like cells following FACS separation. Our results show that NF-*κ*B is highly activated in large RS cells compared with H cells. Oxidative stress further increased NF-*κ*B activity. Moreover, large RS cells are the main contributors of NF-*κ*B activity in the presence or absence of oxidative stress (Figure 5a).

In order to determine which cells are mainly responsible for the secretion of cytokines, we have sorted L428 cells by FACS into H and RS-like cells subpopulations, before and after oxidative stress. The cytokines IL-6, GM-CSF and TNF-*α* were detected under our experimental conditions. Large RS cells expressed higher levels of these cytokines compared with H cells for both untreated and H_2_O_2_-treated cells (Figure 5b).

Since IL-6 was previously shown to reinforce senescence,^26^ we focused on IL-6 production. We examined whether NF-*κ*B inhibition will specifically reduce IL-6 secretion in these cells, using bortezomib, curcumin and JSH-23, a specific NF-*κ*B inhibitor ^27, 28^.^29^ Our results show that as expected, curcumin and JSH-23 inhibited the secretion of IL-6 in both H and large RS-like cells for both treated and non-treated cells. Unexpectedly, the proteasome inhibitor bortezomib rather enhanced the production of IL-6 (Figure 5c). L428 cells lack inhibitor of *κ*B (I*κ*B), which is a known target for bortezomib, suggesting that it could increase IL-6 through other pathways.^27^ Taken together, these observations are consistent with our hypothesis that a subset of HRS cells is senescent, and thus contribute to the induction of a proinflammatory program, the SASP.

## Discussion

In this study, we show that large RS-like cells exhibit characteristics of senescent cells. We demonstrate that RS cells in cHL archive biopsies are stained by antibodies against the cell cycle inhibitors p16^INK4a^ and p21^Cip1^, express NF-*κ*B p65 subunit, and are negative for the proliferation marker Ki-67, suggesting that these cells have ceased to proliferate. In HL-derived L428 cells we could demonstrate that the large RS-like cells stained specifically for the senescence marker SA-*β*-gal. Moreover, we could enhance senescence by oxidative stress in these cells as demonstrated by their increased staining for p16^INK4a^, p21^Cip1^, p53 and *γ*H2AX. One of the hallmarks of senescence is secretion of SASP, driven by NF-*κ*B. Indeed, we show that NF-*κ*B activity and cytokine secretion was elevated in large RS-like cells and inhibited by NF-*κ*B inhibitors.

HL represents a tumor model in which the microenvironment strongly impacts cancer pathogenesis. A crosstalk exists between the tumor cells and the reactive infiltrating cells, which are considered an essential component of the tumor; HRS cells are dependent on anti-apoptotic and pro-survival signals from the microenvironment. One of the hallmarks of the disease is constitutive activation of the NF-*κ*B pathway that provides the tumor cells with strong prosurvival signals. Indeed, we show the presence of phosphorylated nuclear NF-*κ*B p65 in RS cells of HL biopsies (Figure 1). Activation of NF-*κ*B is achieved via multiple mechanisms, that is, mutations of the NF-*κ*B signaling constituents, JAK/STAT pathways and signaling via the TNF receptor superfamily, tyrosine kinases and cytokine receptors.^30^

Senescent cells were previously shown in human skin nevi,^31^ and in tumor cells undergoing radiation or chemotherapy,^32, 33^ but were not described in primary untreated tumors. By defining RS cells as senescent, our studies demonstrate for the first time, to our knowledge, the occurrence of senescent cells in a primary, untreated, tumor mass with relevance for the pathogenesis of the disease. The morphology of RS cells, being large and often multinucleated, is consistent with the large cell morphology characteristic of cells in senescence.^34^ HL is known as a cytokine-producing tumor. Our results with HL-derived L428 cells show that the large RS-like cells are the main cytokine secretion contributors. Secretion of cytokines and inflammatory mediators by HL is in agreement with SASP production by senescent cells.

Cellular senescence pathways are believed to have multiple layers of regulation. Among the cellular pathways reported to regulate senescence are the p16^INK4a^ /pRB pathway, the p19ARF/p53/ p21^Cip1^/WAF1 pathway and the PTEN/p27^Kip1^ pathway.^35, 36^ p16^INK4a^ and p21^Cip1^ have direct inhibitory actions on the cell cycle machinery and correlate well with declining growth rates in cultures undergoing senescence. These cell cycle inhibitors, p16^INK4a^ and p21^Cip1^, were strongly stained in RS cells in most biopsies examined (Table 1; Figure 1). Notably, p16^INK4a^ was recently shown to be a reliable marker of senescence *in vivo*.^37^ Increased expression of the cell cycle inhibitors p16^INK4a^ and p21^Cip1^ cause cell cycle arrest in senescent cells, in line with the fact that RS cells in HL biopsies stained negative for the proliferation marker Ki-67 in most biopsies (Table 1,Figure 1). In order to determine the clinical significance of senescent RS cells, and their correlation with prognosis, a study encompassing a large number of biopsies is required. A step in this direction was taken by Calio *et al.*^38^ who have recently determined the independent prognostic significance of p16^INK4a^ and p21^Cip1^ high expression in HL biopsies. In their study, which encompasses a large number of biopsies, the high expression of either of these two molecules, especially when co-expressed, was found to be of positive prognostic significance.

We also show high expression of p16^INK4a^ and p21^Cip1^ in RS-like cells in L428 cells, and their expression was elevated in response to oxidative stress (Figure 4). These cells were also not proliferative in cell culture (Figure 3c). Furthermore, other markers associated with senescence, such as *γ*H2AX and p53 (Supplementary Figure S1) were basally detected in L428 cells and their expression was increased following oxidative stress.

Senescent cells express SA-*β*-gal, which partly reflects the increase in lysosomal mass.^22^ Early experiments with HL cell lines revealed that large RS cells have no proliferative and clonal growth potential.^16^ Our results show that L428 cells contain a low percentage of spontaneously formed senescent large RS-like cells (Figure 2), that are also SA-*β*-gal positive (Figure 3). The percentage of senescent cells identified by SA-*β*-gal staining is significantly increased in response to oxidative stress (Figure 3). Recently, Rengstl *et al.*^4^ have addressed the important question of how large RS cells evolved from dividing mononucleated Hodgkin cells. They have determined by long-term time lapse microscopy and single-cell tracking that HL cell lines (including L428 and KMH2 cells) contain a rare population of long-lived non-dividing large cells that are generated by re-fusion of small mononuclear progenitors (constituting about 70% of the RS-like cell population). Another mechanism that could be effective in the formation of RS senescent cells is shortening of telomers, shown to occur in RS cells.^39^ Interestingly, it was recently suggested that expression of the endogenous fusogen, ERVWE1, caused cell fusion in normal and cancer cells, leading to the formation of hyperploid syncytia exhibiting features of cellular senescence. Infection by the measles virus, which leads to cell fusion, also induced cellular senescence in normal and cancer cells.^40^

NF-*κ*B was shown to be a master regulator of SASP by influencing the expression of NF-*κ*B target genes. Proteomic analysis of senescent chromatin identified the NF-*κ*B subunit p65 as a major transcription factor that accumulates on chromatin of senescent cells.^25^ Components of the senescence secretome reinforce cell cycle arrest and contribute to tumor suppression by signaling and recruiting components of the immune system.^41^ We show the secretion of the senescence associated cytokines IL-6, GM-CSF and TNF-*α*; large RS cells expressed higher levels of these cytokines compared to H cells for both untreated and H_2_O_2_-treated cells. We have directly demonstrated activation of NF-*κ*B in both small cells and in large RS-like cells. Notably, NF-*κ*B activity and cytokine production, especially IL-6, was significantly higher in large RS cells (Figure 5). We show that curcumin and the NF-*κ*B inhibitor JSH-23 inhibited IL-6 secretion from both H and large RS-like cells as predicted. JSH-23 directly blocks the nuclear localization signal of p65 preventing its accumulation and localization in the nucleus.^29^ Surprisingly, bortezomib, a proteasome inhibitor enhanced IL-6 secretion (Figure 5). Bortesomib inhibits NF-*κ*B by preventing I*κ*B degradation, resulting in its retention in the cytoplasm. Since L428 cells lack I*κ*B, this treatment was ineffective. We suggest that in these cells, IL-6 secretion maybe inhibited through other pathways, independent of the proteasome.^27^ This may explain why bortezomib was not effective in inhibiting IL-6 secretion in L428 cells and failed as treatment for some HL patients.^42, 43^ Although, the idea of using bortezomib as an NF-*κ*B inhibitor in the clinic has failed so far, nevertheless, better alternative NF-*κ*B inhibitors have the potential to be successful in treating HL.

In conclusion, senescent RS cells might support tumor survival and expansion by producing cytokines and chemokines and promoting cellular interactions with other immune cells, shaping the HL microenvironment and supporting the proliferation of H cells.

## Materials and Methods

### Cell culture, antibodies and reagents

HL-derived L428 and KMH2 cells were grown in suspension in RPMI 1640 supplemented with 100 U/ml penicillin, 0.1 mg/ml streptomycin, 2 mM L-glutamine and 10% fetal calf serum (FCS), in a 5% CO2 humidified atmosphere at 37 °C. For the induction of senescence cells were treated with 50 *μ*M H_2_O_2_ for 2 h in RPMI without FCS, washed with complete medium and grown for 4–7 days.

Antibodies used included: anti-p21 ^Cip1^ (sc-397), anti-p16 ^INK4a^ (sc-1207), anti-p53 (sc-126) and anti-p65 (sc-8008) and phospho-NF-*κ*B p65 (Ser 536) (sc-33020- R) which were purchased from Santa Cruz Biotechnology (Santa Cruz, CA, USA), anti-γH2AX phosphor -S139 (ab18311, Abcam, Cambridge, MA, USA), anti-trimethyl Histone H3 (Lys4/Lys9) (Millipore 07-992). Anti CD15 LeuM1 and anti-CD30 BerH2 (Dako, Copenhagen, Denmark), Ki-67, (ab15580, Abcam). For florescence detection the following secondary antibodies were used: donkey anti-rabbit IgG (DyLight 549, 711-505-152, Jackson ImmunoResearch Laboratories, West Grove, PA, USA), Alexa flour-488 conjugated goat anti-mouse (Molecular Probes Inc., Eugene, OR, USA).

### NF-*κ*B luciferase-reporter gene assay

L428 cells stably expressing the luciferase- NF-*κ*B reporter gene were generated as previously described.^18^ For the induction of senescence, cells were incubated with 50 *μ*M H_2_O_2_ for 2 h in RPMI without FCS, washed with complete medium and grown for 96 h. Cells were then FACS sorted according to FSC and SSC, forward- and side-scattering parameters using the FACS, SY3200 (Sony) sorter equipped with 808 nm, and 830 nm solid-state lasers (for FSC and SSC, respectively). Sorted small (H) and large (RS-like) (10^6^ per well) were collected the next day, lysed and assayed using the luciferase reporter kit (Promega, Madison, WI, USA) according to the manufacturer's instructions. Measurements were carried out using a luminometer at 300 nm. Data were normalized to the protein concentration in each lysate as measured by Bradford method (BioRad, Hercules, CA, USA).

### *β*-galactosidase staining

Cells were stained with the nuclear dye, Hoechst, (10 *μ*g/ml, Sigma, St. Louis, MO, USA) in culture medium for 30 min at 37 °C, 5% CO_2_, prior to cytospin. Then, cells were attached to glass slides (26 × 76 mm) by cytospin centrifugation (Shandon Cytospin 4, Ramsey, MN, USA) at 900 r.p.m. for 5 min (70 000 cells per slide). Cells were fixed in 0.5% glutaraldehyde (Sigma), for 20 min at room temperature, washed once with PBS, and kept in PBS at 4 °C.

SA-*β*-gal activity was determined using a previously described protocol^19^ with some modifications. Briefly, cells were washed once with PBS, fixed with 0.5% glutaraldehyde (PBS (pH 7.2)), and washed in PBS (pH 7.2) supplemented with 1 mM MgCl_2_. Cells were stained in X-gal solution (1 mg/ml X-gal (Boehringer, Ingelheim am Rhein, Germany), 0.12 mM K_3_Fe [CN]_6_, 0.12 mM K_4_Fe[CN]_6_, and 1 mM MgCl_2_ in PBS at pH 6.0) overnight at 37 ^°^C. Cells were photographed using an IX70 Olympus optical light microscope (Tokyo, Japan). In order to estimate total cell numbers, cell cultures were stained with Hoechst 10 *μ*g/ml (Calbiochem, HO 33342) for 30 min at 37 °C prior to SA-*β*-gal staining. SA-*β*-gal positive cells were calculated as the percentage of Hoechst-stained cells. The percentage of strongly positive *β*-gal stained large L428 cells compared with small cells was quantified by counting 500 cells in three different experiments.

The determination of SA-*β*-gal activity by flow cytometry was performed as previously described.^20^ Cells were washed once with PBS, and incubated with RPMI supplemented with 0.1 *μ*M Bafilomycin A1 (Sigma,) for 1 h at 37 ^°^C, 5% CO_2_. The substrate for the enzyme, 0.5 mM 5-dodecanoylaminofluorescein di- *β*-D-galactopyranoside (C_12_FDG; Molecular Probes, Invitrogen, Carlsbad, CA, USA) was added to the cells for 1 h at 37 ^°^C (5% CO_2_). The cells were then washed with cold PBS and analyzed using FACS Canto 2 flow cytometer equipped with DIVA 6 software.

### CFDA-SE labeling

Carboxyfluorescein diacetate succinimidyl ester (CFDA-SE) fluorescent staining is used for long-term cell tracking and quantitation of proliferation, both *in vivo* and *in vitro*. Cells were stained with CFDA-SE as previously described.^21^ Briefly, cell pellets were washed three times with PBS, and 1 × 10^7^ cells were incubated in 1 ml of 1 *μ*M CFDA-SE labeling solution for 8 min at 37 °C. After incubation, the labeling reaction was stopped by adding an equal volume of FCS for 1 min. The cells were washed twice with PBS and centrifuged at 1000 r.p.m. for 5 min at room temperature. The cells were then resuspended and incubated in complete RPMI medium for 96 h. The labeled cells were also stained with the nuclear dye, Hoechst, and attached to slides by cytospin (as described above). The percentage of fluorescent positive large L428 cells compared with small cells was quantified by counting 500 cells in three different experiments.

### Cells were photographed using Olympus confocal microscope.

#### Immunohistochemistry

Patient's biopsies of classical HL were obtained and studied by immunohistochemistry at the Institute of Pathology at the Soroka University Medical Center by two expert Hematopatologists (DB and VD). We employed the ABC-peroxidase complex method. In each case, the antibodies were used on conventionally fixed, paraffin-embedded tissue sections as previously described.^22, 23^ Staining was measured semi-quantitatively as specified: −, no positive cells; +, at least 20% strongly positive cells or >30% weakly positive cells.

Staining of slides prepared by cytospin were fixed with 4% paraformaldehyde (Sigma) were incubated for 30 min at room temperature in blocking solution, containing 4% FCS, 0.1% Triton X-100 in PBS. Incubation with the primary antibody was performed at room temperature for 2–12 h in a humidified environment. Cells were washed three times with PBS and then incubated with the fluorescent secondary antibody for 1 h under the same conditions (covered from light). Cells were stained with the nuclear dye Hoechst before cytospin as described above, or by DAPI (4',6-diamidino-2-phenylindole; Sigma) for 15 min after incubation with the fluorescent secondary antibody. The percentage of fluorescent positive large L428 cells compared with small cells was quantified by counting 500 cells in three different experiments. Cells were photographed using an Olympus confocal microscope.

#### Cytokines analysis

For the induction of senescence, cells were incubated with 50 *μ*M H_2_O_2_ for 2 h in RPMI without FCS, washed with complete medium and grown for 96 h. Cells were then FACS sorted as described above. Sorted small (H) and large (RS-like) cells were seeded (1 × 10^6^ per well) in fresh medium and supernatants were collected after 24 h. The secreted cytokines IL-6, TNF-*α* and granulocyte-macrophage colony-stimulating factor (GM-CSF) were detected by ELISA using the Human Multi-Analyte ELISArray Kit (QIAGEN, Hilden, Germany). These three cytokines were quantified using individual ELISA kits. Human IL-6 (eBioscience, San Diego, CA, USA) TNF-*α* (Diaclone Cat. No. 950.090.048) and GM-CSF (Diaclone Cat. No. 873.040.048). IL-6 secretion in response to NF-*κ*B inhibition was determined in supernatants of sorted H and RS-like cells incubated with/without curcumin (30 *μ*M; Sigma), bortezomib (10 nM) (Sigma), or the specific NF-*κ*B inhibitor JSH-23 (100 *μ*M), (Sigma).

## Figures and Tables

**Table 1:** Immunohistochemistry of classical Hodgkin's lymphomas cases

	***n***	***%***
*Gender*		
Female	6	37.5
Male	10	62.5
		
*Age*		
>50	10	37.5
<50	6	62.5

*Type*		
NS	7	43.7
MC	9	56.2
		
CD15	+11	68.75
	−5	31.2
CD30	+14	87.5
	−2	12.5
p16INK4a	+14	87.5
	−2	12.5
p21Cip1	+7	87.5
	−1	12.5
p65	+6	75
	−2	25
Ki-67	+2^a^	25
	6	75

Abbreviations: MC, Mixed cellularity; NS, Nodular sclerosis

Staining was measured semi-quantitatively as specified: –, no positive cells; +, at least 20% strongly positive cells or >30% weakly positive cells

a 20–30% of RS cells were positive and the rest were negative

## Abbreviations

HL: Hodgkin's lymphoma
RS cells: Reed–Sternberg cells
SA-*β*-gal: senescence-associated *β*-galactosidase
SASP: senescence-associated secretory phenotype
NF-*κ*B: nuclear factor *κ*B
IL-6: interleukin 6
TNF-*α*: Tumor necrosis factors *α*
GM-CSF: granulocyte-macrophage colony-stimulating factor
HRS cells: Hodgkin and Reed–Sternberg cells
CFDA-SE: carboxyfluorescein diacetate succinimidyl ester
I*κ*B: inhibitor of *κ*B
