# Supplementary figures and images for: Reed-Sternberg cells in Hodgkin's lymphoma present features of cellular senescence
Source: Cell Death Dis. 2016 Nov 10;7(11):e2457–. doi: 10.1038/cddis.2016.185 (PMC5287295; doi:10.1038/cddis.2016.185)

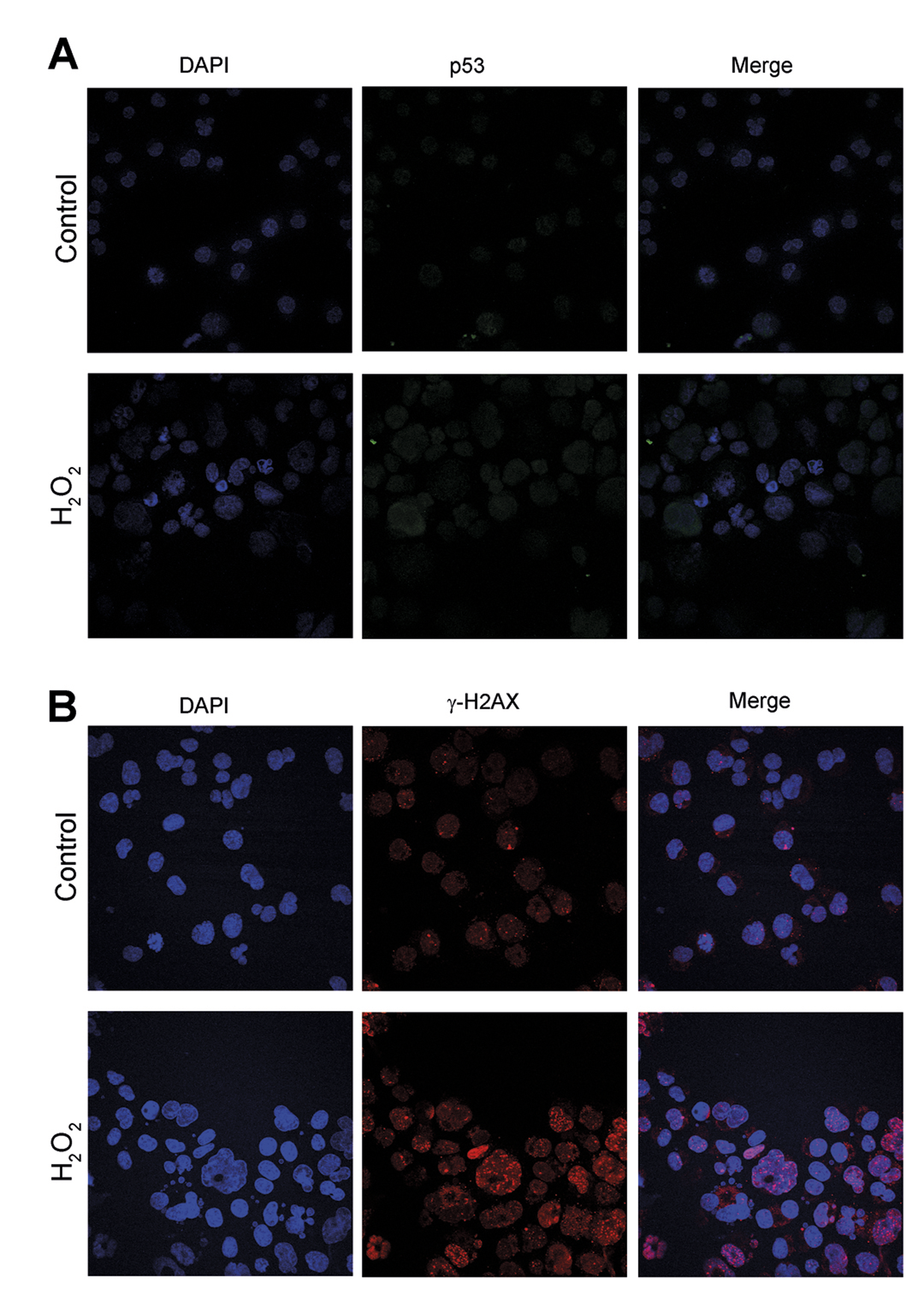

Supplement: Supplementary Information [file cddis2016185x1.tif]
